# Liquid-gas phase-change nanoplatforms for ultrasound-mediated cancer theranostics^☆^

**DOI:** 10.1016/j.ultsonch.2025.107719

**Published:** 2025-12-11

**Authors:** Jin Lei, Jing Lin, Peng Huang

**Affiliations:** Guangdong Key Laboratory for Biomedical Measurements and Ultrasound Imaging, Marshall Laboratory of Biomedical Engineering, International Cancer Center, Laboratory of Evolutionary Theranostics (LET), School of Biomedical Engineering, Shenzhen University Medical School, Shenzhen University, Shenzhen, Guangdong 518055, China

**Keywords:** Ultrasound, Phase-change contrast agents, Nanoplatforms, Perfluorocarbon, *Cancer* theranostics

## Abstract

Ultrasound has emerged as a powerful modality for cancer theranostics due to its noninvasiveness, deep-tissue penetration, and real-time feedback. However, the limited stability and short circulation time of traditional microbubble contrast agents restrict their clinical potential. Liquid-gas phase-change nanoplatforms, also known as phase-change contrast agents (PCCAs), offer a promising alternative by enabling acoustic-triggered vaporization of perfluorocarbon (PFC) cores, facilitating enhanced imaging performance and spatiotemporally controlled therapeutic functions. This review introduces the fundamental mechanisms underlying the liquid–gas phase transition, including acoustic cavitation, heating, and radiation force effects. It then summarizes the design strategies for PCCAs, covering PFC core selection (from low- to high-boiling-point types), diverse carrier platforms (lipid-, polymer-, and nanoemulsion-based systems), and surface functionalization approaches for targeted delivery and stimulus responsiveness. Subsequently, the applications of PCCAs in ultrasound-guided cancer theranostics are discussed, encompassing their roles in diagnostic imaging as well as in ultrasound-mediated mono- and combination therapies, such as chemotherapy, phototherapy, and immunotherapy. Finally, the review concludes with current challenges and future perspectives on smart material integration, multimodal imaging, and precision delivery strategies, which will be key to unlocking the full potential of PCCAs in cancer diagnosis and treatment.

## Highlights

• The design principles and phase-transition mechanisms of phase-change contrast agents (PCCAs) for ultrasound mediated tumor theranostics.
• Recent advances in PCCAs enabling real-time imaging and synergistic therapies.
• Current challenges and future directions toward clinical translation of PCCAs.

## 1 Introduction

Ultrasound, referring to sound waves with frequencies above 20 kHz, has garnered substantial attention as a versatile platform for cancer theranostics due to its inherent advantages, including non-invasiveness, real-time imaging capability, deep tissue penetration, and favorable safety profile [1]. Nonetheless, conventional microbubble-based ultrasound contrast agents are constrained by several intrinsic limitations, such as their micron-scale dimensions, poor extravasation, limited circulation half-life, and susceptibility to acoustic and physiological degradation [2]. These factors collectively hinder their efficacy in tumor-targeted delivery and imaging, particularly in solid tumors characterized by abnormal vasculature and dense extracellular matrices. To overcome these bottlenecks, phase-change contrast agents (PCCAs) have emerged as a next-generation solution. Constructed from PFC cores encapsulated within nanoscale carriers, these agents exploit acoustic trigger to mediate a rapid phase transition from liquid to gas, thereby generating echogenic microbubbles in situ [3]. This phase transformation not only amplifies ultrasound contrast but also endows the system with spatiotemporal control, facilitating precision imaging and stimuli-responsive therapy [4]. The nanoscale PCCAs further enables enhanced permeability and retention (EPR)-mediated tumor accumulation, and provides a flexible platform for functional modifications [5].

Advances in nanotechnology and biomaterials engineering have enabled the rational design of PCCAs with customizable phase-transition thresholds, structural diversity, and therapeutic integration. A variety of design strategies have been explored, encompassing the selection of low-, medium-, and high-boiling-point perfluorocarbons (PFCs), the construction of lipid-, polymer-, or nanoemulsion-based architectures, and the incorporation of molecular ligands for active targeting or microenvironment responsiveness [6], [7], [8], [9], [10]. Beyond diagnostic applications, PCCAs are increasingly being explored as therapeutic platforms capable of co-delivering therapeutic payloads under real-time ultrasound imaging guidance, thereby enabling synergistic interventions such as chemotherapy, phototherapy, and immunotherapy [11], [12], [13], [14]. This multifunctional capability not only enhances treatment precision and spatiotemporal control, but also significantly improves therapeutic efficacy while minimizing off-target effects. Despite rapid progress, a systematic synthesis that correlates the physicochemical design parameters of PCCAs with their acoustic activation behaviors and therapeutic functionalities remains lacking. Previous reviews have primarily focused on either material formulation or biomedical application in isolation, without integrating these aspects into a unified framework.

**Fig. 1:**
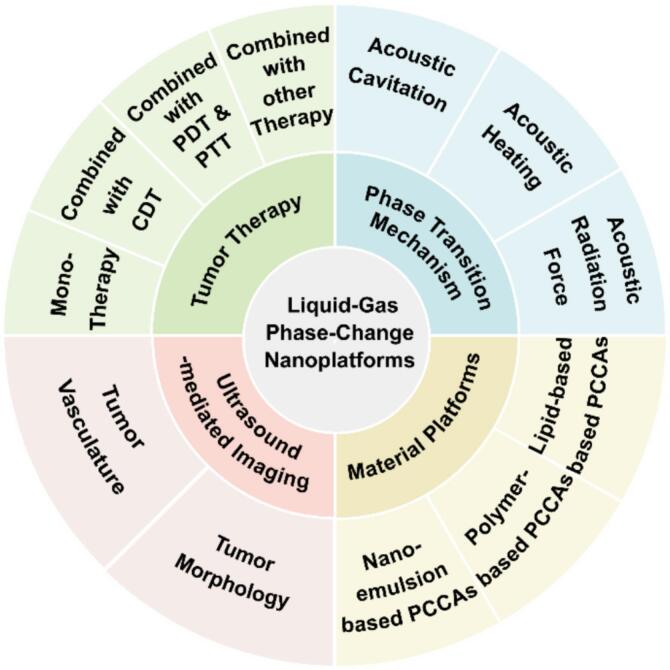
Schematic Illustration of PCCAs for liquid-gas phase-change nanoplatforms.

To address this gap, this review aims to provide an integrated perspective that bridges the materials-acoustics-therapy continuum of PCCAs. By doing so, it delineates the design-to-function relationship that underpins their translation toward ultrasound-mediated cancer theranostics. In this review, a comprehensive overview of liquid-gas phase-change nanoplatforms for ultrasound-mediated tumor theranostics is provided (Fig. 1). The review commences with the elucidation of the fundamental mechanisms and the systematic outlining of the design principles of PCCAs. These principles encompass core material selection, nanostructure engineering, and functional modification strategies. Furthermore, recent advances in the application of these technologies to ultrasound imaging and therapeutic paradigms are highlighted. Finally, the discussion moves on to the current limitations of the technology and the proposal of future directions for the development of clinically translatable, multifunctional PCCA systems that aim to bridge the gap between diagnostic precision and therapeutic efficacy, thus opening new avenues for noninvasive, ultrasound-mediated cancer treatment.

## 2 Mechanistic fundamentals of the liquid-gas phase transition mechanism

**Fig. 2:**
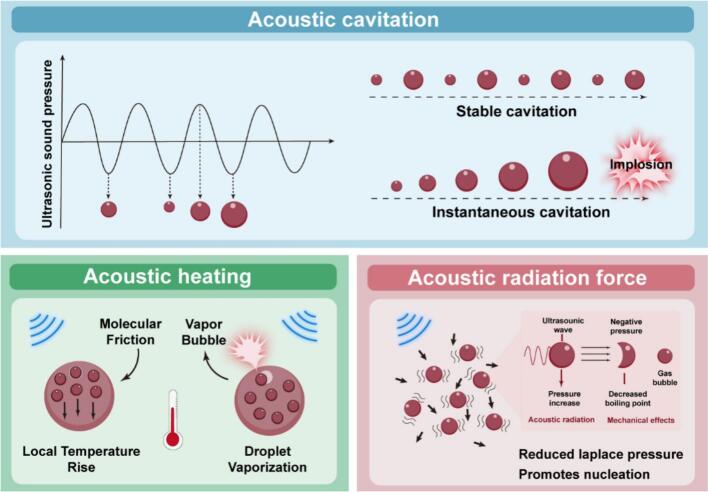
Schematic illustration of the synergistic acoustic mechanisms underlying the liquid–gas phase transition of PCCAs.

Under ultrasonic excitation, nanodroplets can undergo a liquid-gas phase transition into microbubbles with significant echo signals and mechanical activity. This phase transition process is critical for enhancing ultrasound imaging and realizing targeted therapeutic responses [15]. However, the process is not driven by a single physical mechanism, but rather is the result of multiple acoustic effects acting in concert. The three main mechanisms currently recognized include: acoustic cavitation, acoustic heating, and acoustic radiation force and mechanical effects [16] (Fig. 2). Although these three effects are often described as parallel acoustic effects, their contributions to ultrasound-triggered liquid-gas phase transition are inherently hierarchical. Acoustic cavitation serves as the primary driving mechanism because the rapid oscillation and collapse of nuclei generate extreme localized pressure and temperature spikes that directly initiate vaporization. In contrast, acoustic heating functions as a gradual energy accumulation process that elevates the local temperature and reduces the vaporization threshold of perfluorocarbon cores. Acoustic radiation force represents a secondary mechanical effect, facilitating droplet displacement, deformation, and nucleation site formation, thereby assisting but not dominating the phase-transition process. Recognizing this hierarchy is essential for understanding how these acoustic effects act synergistically to regulate the vaporization behavior of PCCAs.

### 2.1 Acoustic cavitation

Acoustic cavitation serves as the primary driving force for ultrasound-induced liquid-gas phase transition due to its ability to generate extreme mechanical and thermal conditions. The effect refers to the physical process of formation and dynamic evolution of tiny bubbles (i.e., cavitation nuclei) induced in a localized region of a liquid under the action of ultrasound waves due to periodic acoustic pressure changes. Essentially, this is due to the alternating positive and negative pressures as the acoustic wave propagates through the liquid: in the negative pressure stage, if the local pressure is below the tensile strength threshold of the liquid, the liquid will undergo local fracture, forming bubble nuclei; and in the subsequent positive pressure stage, these cavitation nuclei are rapidly compressed, and even undergo violent collapse [17]. The implosion of the cavitation bubbles releases extreme local environmental conditions, such as transient high temperatures up to about 5000 K and high pressures in excess of 1000 atm, accompanied by the generation of intense mechanical and chemical effects, including shock waves, microjets, and the generation of reactive radicals (e.g., hydroxyl radicals –·OH) [18]. These effects are not only the core driving mechanism in the ultrasonic phase transition process, but also provide a multidimensional functional basis for its biomedical applications.

Based on the dynamic characteristics of bubbles, ultrasonic cavitation can be further classified into stable cavitation and transient cavitation. Stable cavitation refers to the oscillatory state of bubbles maintained under the cyclic action of ultrasound waves, which mainly induces low-intensity fluid shear and helps to enhance the exchange of substances and cell membrane permeability, and thus is widely used in scenarios such as drug delivery and gene transfection [19]. In contrast, transient cavitation occurs when a bubble undergoes a brief expansion and then rapidly collapses, accompanied by high-intensity physical destruction effects, such as high-speed microjets (up to ∼ 100 m/s) and extreme thermodynamic conditions, which can effectively destroy cellular structures and promote the formation of reactive oxygen species, and is suitable for therapeutic strategies with a high demand for destruction, such as tumor ablation [20], [21]. It should be noted that the occurrence and intensity of the cavitation effect are synergistically regulated by a variety of physical parameters, including the viscosity of the liquid, the solubility of the gas, and acoustic parameters such as the frequency, intensity, and duration of action of the ultrasound [22]. By precisely regulating these conditions, the modulation of cavitation behavior can be achieved to tailor the design and optimization of different biomedical application strategies, such as targeted drug release, ultrasound imaging enhancement, and localized thermal/chemotherapeutic treatments.

### 2.2 Acoustic heating

Acoustic heating contributes to PCCAs vaporization by providing localized thermal energy that lowers the phase-transition threshold of perfluorocarbon cores [23]. The basic mechanism lies in the microscopic mechanical vibrations that occur within the tissue under the action of acoustic waves. Although ultrasound is an elastic wave, when propagating in real media, the vibration process is not an ideal dissipation-free process, but is accompanied by significant energy dissipation, which is mainly manifested as internal friction caused by the relative displacement between the liquid phase and the solid phase components within the tissue [15]. This micro-scale “pulling” effect can cause irreversible energy conversion into heat, leading to a local temperature increase. This thermal effect provides the critical heat for liquid-gas phase change materials (e.g., PFCs) to realize the phase change from the liquid phase to the gas phase.

The bubbles generated during this phase change process not only significantly enhance the scattering ability of ultrasound, increase the echo signal intensity and achieve enhanced imaging contrast, but also act on the intercellular junctions and basement membrane structure through mechanical perturbation to temporarily increase the permeability of local tissues, thus facilitating the penetration and diffusion of drugs or diagnostic probes [24]. Therefore, the liquid-air phase transition mechanism inspired by the thermal effect of ultrasound plays an irreplaceable role in the construction of an integrated tumor diagnostic and treatment platform, which not only significantly improves the sensitivity and resolution of ultrasound imaging, but also provides a precise means of physical triggering for tumor microenvironment-responsive drug release.

### 2.3 Acoustic radiation force and mechanical effects

Acoustic radiation force and mechanical perturbation facilitate phase transition by inducing droplet displacement, deformation, and nucleation instability. Ultrasound Radiation Force (URF) is a directional constant force generated by the acoustic pressure gradient during the propagation of sound waves, which can be applied to tiny particles or liquid droplets in the medium [25]. This force can effectively drive droplets to displace, collide, and converge on surrounding structures on a microscopic scale, resulting in significant shear stresses and pressure concentrations in a localized region [26]. These mechanical perturbations can destabilize the internal mechanics of the droplet, inducing instability in the liquid core and driving its transition to the gaseous state [27].

At the same time, ultrasonic waves, as a kind of longitudinal mechanical waves, will exert continuous compression and tension on the droplet during the propagation process, and this kind of periodic mechanical perturbation puts the core of the droplet in a kind of “sub-stable state” or “overheating state” [28]. In this state, the droplet is prone to form a nucleation site. Once the nucleation site is formed and obtains enough energy, it will expand rapidly and drive the droplet to undergo a drastic liquid-gas phase transition [29]. After the phase transition is completed, the droplet is transformed into bubbles, which have strong ultrasound scattering and reflecting ability due to their significant acoustic impedance difference, significantly enhancing the ultrasound contrast signal and providing a strong support for ultrasound imaging-guided tumor visualization and treatment [30].

In liquid-gas phase transition nanoplatforms, ultrasound induces phase transitions through a variety of mechanical and thermal mechanisms. Among them, radiative force and mechanical perturbation provide continuous deformation and stress accumulation, while stable cavitation further triggers microfluidic perturbation, which together lower the energy barrier of phase transition; when the energy accumulation is sufficient or the acoustic intensity increases, the instantaneous cavitation burst energy can instantaneously penetrate the liquid droplet to complete the phase transition; and the thermal effect serves as a means of regulating and controlling the threshold of vaporization by increasing the temperature. The four effects can be selected or used synergistically to realize a controlled and efficient ultrasound-induced phase transition process. Moreover, mechanistic advances that exploit piezotronic and piezo-phototronic effects reveal additional routes to enhance sonosensitizer activity and acoustic-to-chemical energy transduction, offering design principles relevant to PCCAs-based SDT systems [31].

## 3 Engineering and design strategies for PCCAs for ultrasound

Building upon the fundamental mechanisms of liquid-gas phase transition discussed above, including acoustic cavitation, acoustic heating, and acoustic radiation force, the rational design of PCCAs requires precise control over their physicochemical characteristics to achieve predictable acoustic responses. The selection of core materials determines the threshold and reversibility of vaporization, the carrier matrix governs droplet stability, biocompatibility, and acoustic energy transfer, and surface functionalization enables targeted accumulation as well as stimuli-responsive activation within the biological environment. A comprehensive understanding of these aspects is therefore essential for tailoring PCCAs toward specific imaging or therapeutic objectives.

In this section, the major design strategies for PCCAs are summarized according to three interrelated dimensions: (1) Core Materials, focusing on the phase-change characteristics of perfluorocarbon cores; (2) Carrier Platforms, highlighting how lipid-, polymer-, and nanoemulsion-based architectures modulate stability and acoustic behavior; and (3) Functionalization, emphasizing approaches that impart targeting capability and stimulus responsiveness under physiological conditions.

### 3.1 Core materials (PFCs)

**Fig. 3:**
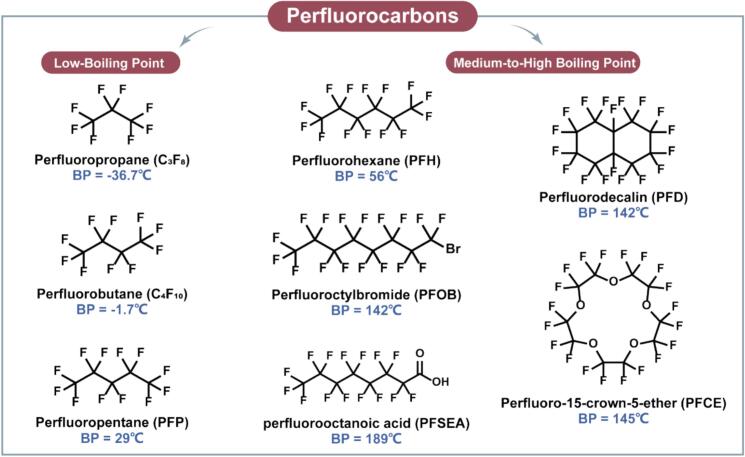
Structures of the PFCs commonly used for formulating PCCAs.

PFCs are widely used as core materials in the current research and application of liquid-gas PCCAs due to their good chemical inertness, biocompatibility, and easily controllable phase-change behavior. The boiling point of PFCs is affected by the length of carbon chains and the number of branched chains in their molecular structure, and can be broadly categorized into three types: low boiling point PFCs (<40°C), medium boiling point PFCs (40–70°C), and high boiling point PFCs (>100°C) (Fig. 3 and Table 1). PFCs with different boiling points exhibit differentiated physicochemical properties and can be strategically selected for specific ultrasound imaging and therapeutic needs (Table 2).

**Table 1:** Representative PCCA platforms and design-function comparison.

PFC Core	Boiling point (℃)	Carrier Type	Functionalization	Application	Refs
C_3_F_8_, C_4_F_10_	−36.7°C	DSPC, DSPE-PEG_2K_	\	Blood-brain permeabilization	[33]
PFP	29°C	DSPC, DSPE-PEG_2K_, Cholesterol	PD-L1-targeted	Early diagnosis of PDAC	[34]
		DOPE-DMA, DPPC, DSPE-PEG_2K_	pH-responsive	Active tumor penetration	[5]
PFB	29°C	TLNs@C/R	MMP-2-sensitive	Intratumor aggregation, sonodynamic immunotherapy	[9]
PFH	56°C	OCMC	pH-responsive	Tumor CEUS imaging	[36]
		DPPC, DSPE-PEG_2K_, Cholesterol	\	Diminishing CSC populations	[44]
		DPPC, DSPE-PEG-SH, Cholesterol, DOTAP	cRGD-modified	Tumor ferroptosis, PTT	[45]
		DPPC, DSPE-PEG_5K_, DPPA	PD-L1-targeted	anti-PD-L1 mAb therapy	[46]
PFCE	146°C	PEG-PDLA, PEG-PLLA, PEG-PCL	\	Ultrasound imaging, ^19^F MRS and MRI	[37]
PFOB	142°C	BSA	\	HIFU therapy	[40]
		Poly(2-oxazoline) block copolymer	GSH-responsive	Nonviral gene delivery	[47]
PFD	142°C	HSA	Mitochondria-targeted	SDT	[41]

**Note:** C_3_F_8_, perfluoropropane; C_4_F_10_, perfluorobutane; DSPC, 1,2-distearoyl-*sn*-glycero-3-phosphocholine; DSPE-PEG_2K_, 1,2-distearoyl-*sn*-glycero-3-phosphoethanolamine-N-[methoxy(polyethylene glycol)-2000]; PFP, perfluoropentane; PDAC, pancreatic ductal adenocarcinoma; DOPE-DMA, 1,2-dioleoyl-snglycero-3-phosphoethanolamine lipid; DPPC, 1,2-dipalmitoyl-*sn*-glycero-3-phosphocholine; PFB, 1,1,1,3,3-pentafluorobutane; OCMC, o-carboxymethyl chitosan; PFH, perfluorohexane; CEUS, contrast enhanced ultrasound; CSC, cancer stem cells; PFCE, perfluoro-15-crown-5-ether; PEG-PDLA, poly(ethylene oxide)–co-poly(D,L-lactide); PEG-PLLA, poly(ethylene oxide)–co-poly(L-lactide); PEG-PCL, poly (ethylene oxide)–co-polycaprolactone; PFOB, perfluorooctyl bromide; HIFU, high intensity focused ultrasound; BSA, bovine serum albumin; PFD, perfluorodecalin; HSA, human serum albumin; PEPP, polyepinephrine; PTT, photothermal therapy.

**Table 2:** Comparison of major PCCA carrier platforms.

Carrier platform	Key features	Advantages	Limitations
Lipid-based PCCAs	Phospholipid shells (e.g., DPPC, DSPE, Chol).	• Excellent biocompatibility. • Easy surface modification and ligand conjugation. • Strong acoustic responsiveness and efficient ADV. • Suitable for co-loading drugs and PFCs.	• Multi-component system increases formulation complexity. • Limited long-term stability (fusion, leakage). • Scale-up may be less reproducible.
Polymer-based PCCAs	PLGA, PEG copolymers, fluorinated polymers; tunable mechanical strength.	• High structural stability and longer circulation. • Large loading capacity for drugs/oxygen/sensitizers. • Good control over vaporization threshold. • Support multimodal imaging.	• Multi-step synthesis may cause batch variability. • Over-crosslinking can reduce nonlinear acoustic response. • Polymer degradation profile requires safety evaluation.
Nanoemulsion-based PCCAs	Protein- or surfactant-stabilized PFC nanoemulsions.	• Simple fabrication and scalable. • High PFC loading and strong ADV effect. • Good biocompatibility when protein-stabilized. • Suitable for oxygen delivery and interface-driven mechanisms.	• Stability depends on protein–PFC interactions. • Risk of size growth or phase separation during storage. • More sensitive to acoustic parameters.

#### 3.1.1 Low-boiling point PFCs

Low-boiling-point PFCs enable highly sensitive ultrasound-triggered vaporization due to their low thermodynamic thresholds, making them suitable for low-mechanical index (MI) imaging and rapid activation [32]. This makes them particularly well-suited for applications requiring high sensitivity or contrast at minimal acoustic energy levels. For instance, Novell et al. investigated two nanodroplet systems based on C_3_F_8_ (boiling point −37°C) and C_4_F_10_ (boiling point −2°C) and systematically compared their vaporization thresholds and acoustic profiles in vitro [33]. C_3_F_8_ nanodroplets initiated vaporization at a MI of 0.24, emitting harmonic signals at 2*f_0_* and 1.5*f_0_* from MI = 0.37 onwards, with broadband noise observed at MI = 0.45. In contrast, C_4_F_10_ nanodroplets demonstrated a higher vaporization threshold of MI = 0.94, with signal emission beginning at MI = 0.53 (2*f_0_*) and MI = 0.73 (1.5*f_0_*), and noise detected at MI = 0.78. These findings suggest that increasing molecular chain length raises the energy threshold for vaporization. In vivo studies further validated these differences. In a blood–brain barrier (BBB) permeabilization model, C_3_F_8_ nanodroplets achieved widespread BBB opening under low-pressure ultrasound, whereas C_4_F_10_ droplets induced localized (∼400  μm) BBB modulation under higher pressure. These results highlight the potential to tailor PFC composition for specific anatomical and pathological contexts.

Beyond diagnostic capabilities, low-boiling PFCs can also enhance therapeutic outcomes. For example, *Chen* et al. constructed a liquid-gas phase transition lipid nanoparticle (aPDL1-DTX/PFP@Lipid) co-loaded with docetaxel (DTX) and PFP co-modified by anti-PD-L1 antibody (aPD-L1) [34]. The nanoparticles underwent a liquid-gas phase transition under low-intensity pulsed ultrasound stimulation to form microbubbles, which significantly enhanced the diagnostic signals of ultrasound molecular imaging and synergistically achieved anti-tumor therapy with tumor growth inhibition rate of 88.91%. In addition, this therapeutic strategy promoted the remodeling of the tumor immune microenvironment while achieving oxygen supply through the release of PFP, effectively alleviating the hypoxic state of the tumor tissue. As a result, this study provides a strong basis for the development of a new strategy for ultrasound-mediated pancreatic ductal adenocarcinoma diagnosis and treatment integration. Despite the therapeutic benefits, the multi-component architecture, which integrating antibodies, chemotherapeutics, lipids, and volatile PFP, may pose challenges for batch-to-batch reproducibility and large-scale translational manufacturing.

However, intrinsic thermodynamic instability of Low-boiling point PFCs also introduces practical limitations. Because these PFCs (e.g., PFP, C_3_F_8_) are close to or below physiological temperature, they may undergo premature vaporization during storage, circulation, or even during formulation, leading to reduced encapsulation efficiency, size polydispersity, and unpredictable in vivo activation. These stability concerns may complicate quality control and hinder scalable manufacturing.

#### 3.1.2 Medium-to-high boiling point PFCs

Medium- and high-boiling-point PFCs offer superior in vivo stability and controlled activation, allowing extended imaging windows and stimulus-responsive therapy. These features make them ideal candidates for applications requiring extended imaging windows or controlled, stimulus-responsive drug release. Among them, perfluorohexane (PFH), a representative PFCs, has been successfully applied to the development of multifunctional nanodroplets [35]. Li et al. constructed PFH nanodroplets (NDs) encapsulated by O-hydroxymethyl chitosan and loaded with hydroxychloroquine (HCQ) for tumor targeting and ultrasound imaging [36]. In vitro ultrasound imaging showed that the NDs showed a peak of echo enhancement within 5 min, followed by attenuation, suggesting that they underwent a liquid-gas phase transition under ultrasound. 30 min after triggering the “Flash” button, the echo intensity increased dramatically (12–49 dB) and disappeared rapidly, suggesting that the NDs were highly sensitive to acoustic stimuli. In vivo experiments further demonstrated that NDs significantly enhanced the imaging signals at the tumor site within 60 s after injection, and the imaging enhancement lasted for more than 180 s. This study demonstrated that PFH-based NDs not only excel in acoustic response imaging, but also have the potential for drug delivery and therapeutic synergism, which provides strong support for ultrasound imaging-guided precision tumor therapy.

High-boiling point PFCs such as perfluoro-15-crown-5-ether (PFCE), perfluorooctanoic acid (PFSEA), perfluorooctyl bromide (PFOB), and perfluorodecalin (PFD) offer additional opportunities for integration into multimodal platforms and oxygen delivery systems [37]. For instance, Sun et al. constructed a ^19^F MRI visualized and LIFU-triggered multimodal therapeutic diagnostic nanoplatform based on PFCE, which, through modification of the epidermal growth factor receptor, enabled the targeting lung delivery and deep penetration of the nanoplatform [38]. *Chen* et al. prepared nanoparticles (THPPpf-COPs) with sonodynamic therapeutic effects based on the PFSEA and sonosensitizer *meso*-5, 10, 15, 20-tetra (4-hydroxylphenyl) porphyrin (THPP), and formed nanotherapeutic agents capable of oxygen-carrying (PFCE@THPPpf-COPs) by PECE loading [39]. After intratumoral injection, the nanoparticles can effectively alleviate hypoxia and achieve a potent tumor killing effect by activating oxygen into reactive oxygen species under the action of ultrasound while inducing ICD. In another study, Yin et al. developed a nanoemulsion for MRI/CT dual diagnosis. They first prepared MnO_2_ with BSA into nanoparticles by biomineralization reaction, and then further prepared it with PFOB into nano-emulsions by ultrasonic emulsification method [40]. The nano-emulsions can be used as a new strategy for tumor-targeted immunotherapy through high-intensity focused ultrasound treatment. Meanwhile, PFD plays an important role in ultrasound-induced tumor therapy. Guo et al. constructed a PIH-NO system by wrapping PFD and acoustic sensitizer IR780 with human serum albumin-based NO donor [41]. Among them, PFD was combined with acoustic sensitizer based on good oxygen-carrying properties to achieve potent acoustic power/immunotherapy effects in 4 T1 tumor model. In addition, high boiling point PFCs can undergo phase transition under external thermal stimulation or localized acoustic thermal effects, which gives them a significant advantage in temperature-activated therapeutic strategies, enabling more precise “on/off” control of the therapeutic process, thus improving therapeutic targeting and safety [42].

Collectively, low-boiling PFCs offer superior acoustic sensitivity but suffer from stability issues, whereas medium/high-boiling PFCs provide predictable activation at the cost of higher energy thresholds. This trade-off suggests that material choice should be application-driven rather than universally optimized. In addition to the intrinsic physicochemical properties of the PFC core, the droplet size of PCCAs also plays a crucial role in determining the vaporization threshold. Smaller nanodroplets exhibit higher Laplace pressure (P*_L_* = 2γ/r), which stabilizes the liquid core and increases the acoustic rarefaction pressure required to initiate acoustic droplet vaporization (ADV) [43]. Consequently, sub-200 nm PCCAs typically demonstrate significantly higher vaporization thresholds compared with larger droplets (e.g., 300–800 nm). As a result, size optimization, together with boiling point and shell architecture, should be considered an integral design parameter when tailoring PCCAs for specific imaging or therapeutic applications.

### 3.2 Carrier platforms (lipid/polymer/nanoemulsion)

To meet the complex demands of imaging-guided tumor therapy, researchers have developed diverse material platforms for PCCAs, enabling multifunctionality, biocompatibility, and precise responsiveness. Among them, lipid-based, polymer-based, and nanoemulsion-based PCCAs have attracted extensive attention due to their structural versatility and functional tunability.

#### 3.2.1 Lipid-based PCCAs

**Fig. 4:**
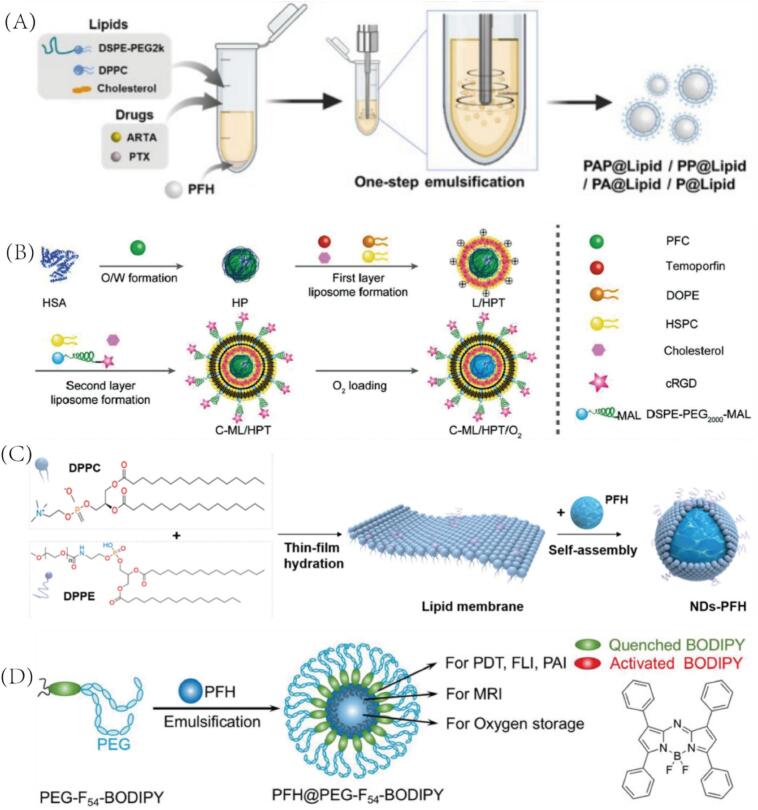
(A) Preparation route of PAP@Lipid. (B) Schematic preparation of C-ML/HPT/O_2_, composed of cRGD (C) Composition of multilayer liposomal (ML), HSA, PFC, temoporfin (T), and oxygen (O_2_). (C) Bioinspired design and preparation of NPs-PFH. (D) Construction of PFH@PEG-F54-BODIPY, which is self-assembled by a BODIPY amphiphile and PFH. Reproduced with permission from Ref.[8], [44], [50], [52] © 2022 WILEY, © 2024 Elsevier, © 2015 Elsevier, © 2019 WILEY respectively.

Lipid-based PCCAs represent one of the most extensively developed platforms due to their excellent biocompatibility, flexibility in structural modification, and strong interaction with cellular membranes. In particular, various phospholipids such as DPPC, DSPE, DPPE, and cholesterol are frequently utilized to construct stable lipid shells [48], [49]. These shells can encapsulate PFC cores and therapeutic agents simultaneously, enabling efficient ultrasound imaging and therapeutic responses. For instance, Chen et al. successfully constructed drug-loaded ultrasound-sensitive nanodroplets (PAP@Lipid) using a one-step emulsification method, realizing the simultaneous encapsulation of two hydrophobic drugs, ARTA and PTX, and superhydrophobic PFH were encapsulated at the same time, thus proposing a new strategy for ultrasound-mediated tumor cancer stem cells modulation based on ultrasound [44] (Fig. 4A). Specifically, the lipid shells of the nanodroplets were formed from DSPE-PEG_2K_, DPPC, and cholesterol in a certain ratio by solvent evaporation method. The amphiphilic nature of the lipid bilayer helps the hydrophobic drug to be embedded in its hydrophobic layer. Subsequently, the addition of PFH to the lipid-drug aqueous solution and ultrasonication not only facilitated the emulsification process, but also enabled the efficient encapsulation of PFH in the lipid structure. The resulting liposome nanodroplets were well dispersed, regularly spherical, and exhibited excellent ultrasound imaging contrast. Building on this strategy, Yan et al. developed a nanoengineering approach to combine liposome-encapsulated PFCs with neutrophils to construct a nano-delivery system for visual diagnosis and treatment of tumors [8]. First, they encapsulated human serum albumin (HSA) in the outer layer of PFC to prevent oxygen leakage, followed by encapsulation using three components, DOPE, HSPC, and cholesterol, on its surface to constitute lipid-based PCCAs, and simultaneously encapsulated the lipid-soluble acoustic sensitizer temoporfin molecule between the phospholipid bilayers (Fig. 4B). Subsequently, the construction of multifunctional nanoparticles was completed by the modification of cRGD on the surface of lipid-based PCCAs, which can be used for real-time monitoring of tumor tissues and effective acoustic therapy by fluorescence imaging and ultrasound imaging. Similarly, Lu et al. prepared lipid shells based on DPPC and DPPE by thin-film hydration method, and encapsulated PFH in them by self-assembly method to prepare nanodroplets with anti-tumor immunotherapeutic effects (Fig. 4C) [50]. The lipids therein improved the stability of PFH in vivo, and at the same time, PFH could significantly reduce the extremely high cavitation threshold required by mechanical high-intensity focused ultrasound (mHIFU) for ablating tumors, prompting the activation of tumor immunity by cellular debris. In another innovative design, Wang et al. designed a nanodroplet consisting of a lipid monolayer containing an organosiloxane surface atomic layer [51]. Among them, perfluorocarbons and the chemotherapeutic drug doxorubicin (DOX) were encapsulated, and var7-DSPE-mPEG_2K_ with tumor pH responsiveness was modified on the surface. Under HIFU, DOX and oxygen in the nanodroplets could be efficiently co-released while enhancing ultrasound imaging, thus realizing imaging-guided drug delivery and tumor therapy. These diverse strategies highlight the versatility of lipid-based PCCAs in integrating imaging and therapeutic functions. Moreover, the tunable physicochemical properties of lipid components provide a foundation for further engineering of responsive or targeted nanodroplets. However, their multilayer architectures and co-loading of drugs, sensitizers, and PFCs may complicate scale-up, control of lipid packing, and maintenance of physical stability during storage and transport.

#### 3.2.2 Polymer-based PCCAs

Poly(lactic-co-glycolic acid) (PLGA), an FDA-approved biodegradable polymer, has been extensively employed in the design of PCCAs due to its excellent biocompatibility, tunable degradation profiles, and capacity for encapsulating hydrophobic perfluorocarbons. Its versatility allows for structural optimization and integration with various functional payloads to support both diagnostic and therapeutic applications. Several studies have demonstrated the feasibility of PLGA-based PCCAs in achieving multimodal imaging, oxygen delivery, and synergistic treatment strategies [53]. For instance, Mangala Srinivas et al. confirmed that PLGA nanoparticles loaded with PFCE have a fractal multinuclear structure by nuclear magnetic resonance spectroscopy and small angle neutron scattering experiments [54]. Also, they used multimodal imaging to confirm that the nanoparticles were stable in ex vivo and in vivo imaging. This suggests that PLGA is a reliable choice for the construction of PCCAs. Subsequently, Li et al. constructed a novel oxygen-loaded mimetic perfluorocarbon nanoparticle (M@P-SOP) by using a complex emulsion method to modify FDA-approved PLGA on PFH, while loading superparamagnetic iron oxide, and encapsulating a tumor cell membrane on its surface by thin film extrusion [55]. The formulation enables complementary multimodal tumor imaging and effective tumor imaging and treatment through HIFU-facilitated combination with immunotherapy. Yet, polymeric shells, especially those requiring crosslinking or multi-step assembly, may exhibit batch variability and introduce challenges in achieving consistent acoustic response thresholds across large-scale production.

Beyond lipids, polymer-based PCCAs provide enhanced mechanical strength and prolonged circulation times, offering another important platform. Liu et al. designed and synthesized a series of amphiphilic copolymerized peptides as shell layer materials for coating PFCs microbubbles [6]. The hydrophilic PEG blocks formed the outer layer of the microbubble shell layer, and the hydrophobic and fluorinated PFOK blocks formed the inner layer. The fluorinated inserts are characterized by low interfacial tension and low Laplace pressure, which contribute to the stability of the microbubbles when interacting with the inner core octafluoropropane (C_3_F_8_). To further enhance the structural stability of the microbubbles, the researchers also synthesized copolymerized polypeptides containing crosslinkable diacetylene groups in the side chains, and induced the crosslinking reaction of the diacetylene groups by UV light, which effectively improved the mechanical stability of the microbubbles. However, the crosslinked structure inhibited the nonlinear oscillatory behavior of the microbubbles, which in turn led to a decrease in the US signal intensity. To overcome this problem, the research team further developed copolymerized peptides with a maleimide (MI) functional group at the end. This material can be covalently coupled with plasma proteins such as albumin via in situ Michael addition reaction to form stable protein-modified microbubbles (MI-MBs). Experiments have shown that MI-MBs significantly enhance their nonlinear oscillating ability while maintaining good stability, thereby enhancing ultrasound imaging signals.

Moreover, Huang et al. designed a poly(ethylene glycol)-boron dipyrromethene amphiphile (PEG-F_54_ BODIPY) with 54 fluorine-19 (^19^F), and the construction of a nanodroplet integrating photodynamic therapy (PDT), FLI, PAI, MRI, and oxygen loading could be realized by this synthesized amphiphilic polymer encapsulating PFH [52] (Fig. 4D). Multiple imaging modalities allow for multilevel and diversified diagnosis of the tumor structure, which further guides the enhanced BODIPY tumor ablation effect under oxygen loading.

#### 3.2.3 Nanoemulsion-based PCCAs

In addition to lipid and polymer systems, nanoemulsions stabilized by natural biomacromolecules provide another effective route for constructing PCCAs, particularly due to their simplicity and tunable surface chemistry. For instance, Ju et al. synthesized macrophage membrane-encapsulated oxygen and drug delivery system (CPIM) using bovine serum albumin as a carrier using ultrasound and extrusion methods. Among them, PFOB was the oxygen-carrying and ultrasound imaging reagent, indocyanine green (ICG) was the photosensitizer, and α-mangostin was the tumor pro-permeability agent. They evaluated the in vivo homologous targeting and hypoxia-relieving ability of CPIM by NIR imaging and photoacoustic (PA) imaging, and also further demonstrated its potential for tumor growth inhibition. In a different approach, Jiang et al. developed a PFCs-based nanoemulsion platform to investigate electron transfer at the liquid–liquid interface under ultrasound stimulation [7]. To improve the aqueous dispersibility and colloidal stability of the nanoemulsion, HSA was employed as a surface modifier. HSA not only enhanced the water solubility of the nanoemulsion but also stabilized its structure under ultrasonic cavitation, thereby promoting the formation of dynamic liquid–liquid interfaces and facilitating efficient electron transfer. Upon ultrasound irradiation, the generation and collapse of cavitation bubbles induced high-frequency liquid–liquid contact between the water phase and the PFC nanoemulsion. This contact enabled electrons to transfer from water molecules and hydroxide ions (OH^−^) to the PFC phase, leading to the production of hydroxyl radicals (·OH), resulting in potent antitumor outcomes. This study highlights a novel liquid–liquid interface-enhanced radical therapy approach and underscores the pivotal role of HSA in constructing multifunctional therapeutic nanoplatforms.

Further advancing this concept, Dmitry A. Gorin and colleagues developed a tri-modal imaging nanodroplet platform via a layer-by-layer assembly strategy [56]. The system employed PFP, with a boiling point of 29°C, as the US imaging core. To enhance stability, a bovine serum albumin (BSA) shell was coated on the PFP core. This BSA layer not only improved the biocompatibility of the nanodroplets but also increased the boiling point of PFP to approximately 47°C under laplace pressure, significantly enhancing the thermal stability and structural integrity of the nanodroplets during in vivo and in vitro applications. Subsequently, Fe_3_O_4_ nanoparticles and ICG were sequentially loaded onto the surface, integrating magnetic resonance imaging, photoacoustic imaging, and ultrasound imaging functionalities into a single platform. Overall, lipid-based PCCAs are easiest to functionalize, polymer-based systems offer highest mechanical robustness, and nanoemulsions provide superior scalability, indicating that carrier selection inherently reflects a balance between stability, responsiveness, and manufacturability.

### 3.3 Functionalization (Targeting/stimulus-responsiveness)

#### 3.3.1 Targeted delivery

To enhance the tumor accumulation and biospecificity of PCCAs, various active targeting strategies have been developed based on the overexpression of disease-specific biomarkers in tumor tissues. Unlike passive accumulation through the EPR effect, active targeting improves delivery precision and therapeutic index [57]. This section introduces several representative approaches employing ligands, antibodies, and engineered proteins to functionalize PCCAs for targeted delivery.

Receptor–ligand interactions are among the most commonly exploited strategies for tumor targeting. For instance, integrin α_v_β_3_ is highly expressed in both liver cancer cells and tumor neovasculature. Dong et al. developed a multifunctional PCCA (ATO/PFH NPs@Au-cRGD) by co-loading PFH and arsenic trioxide into phospholipid nanoparticles and decorating the surface with a gold shell and cRGD via Au-S bonding [45]. This design enabled efficient α_v_β_3_-targeted accumulation while providing ultrasound-triggered imaging enhancement and chemotherapeutic delivery, demonstrating synergistic theranostic capability.

**Fig. 5:**
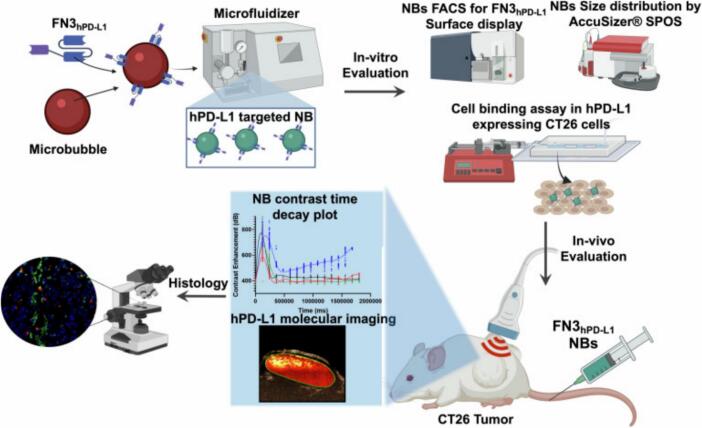
Schematic illustration of the microfluidic fabrication of FN3hPD-L1-NBs targeting hPD-L1, along with their in vitro and in vivo assessments as molecular ultrasound contrast agents. Reproduced with permission from Ref.[45]© 2023 WILEY.

Beyond ligand–receptor targeting, antibody-based strategies enable high specificity in molecular imaging of immune checkpoint proteins. Paulmurugan et al. designed a targeted nanobubble by covalently conjugating an anti-human PD-L1 (hPD-L1) monoclonal antibody onto the surface of liposomes encapsulating PFP [46] (Fig. 5). This nanobubble enabled molecular-level imaging of PD-L1 expression on both intravascular and extravascular tumor cells using contrast-enhanced ultrasound molecular imaging (CE-USMI). Compared to non-targeted controls, this system yielded a ∼ 3-fold increase in in vivo signal intensity, significantly enhancing tumor visualization and providing insights into immune status monitoring.

Engineered PD-1 fusion proteins also enable targeted delivery. Yuan et al. designed an RGD- and mPD1-modified PFH nanodroplet (NDS^mTx^) for LFUS-triggered release. RGD mediates tumor-vascular targeting, and LFUS rupture releases mPD1, which covalently binds and degrades PD-L1, enhancing antitumor immunity [10]. Extending PFC-based PCCAs to other acoustic modes, Gambhir et al. developed ligand-modified nanodroplets that act as ultra-high-frequency RF contrast agents, showing ＞1600-fold stronger signals than Fe_3_O_4_ nanoparticles and enabling GRPR-targeted imaging in prostate cancer [58]. However, ligand-receptor-based strategies often increase immunogenicity risk, require precise ligand density control, and add complexity to manufacturing and regulatory evaluation.

#### 3.3.2 Stimulus responsiveness

In addition to passive accumulation and external acoustic activation, incorporating responsiveness to endogenous tumor microenvironmental cues offers a powerful approach to enhancing the precision and efficacy of PCCAs. These stimuli-responsive systems are engineered to undergo phase transitions or trigger therapeutic payload release in response to internal biochemical triggers such as pH, redox status, enzymes, or hypoxia. This section highlights representative designs of PCCAs that leverage such tumor-specific stimuli to achieve controlled activation, enhanced imaging contrast, and therapeutic synergy.

The acidic TME originates from the Warburg effect, producing excess lactate/protons and lowering extracellular pH to ∼6.5–6.8 (vs. ∼ 7.4 in normal tissues). This pH gradient enables pH-responsive PCCAs to undergo structure/phase changes or trigger payload release selectively in tumors. A representative system is Huang et al.’s pH-responsive liposomal nanodroplet (SCGLN), co-assembled from DOPE-DMA (acid-cleavable), DSPE-PEG, DPPC, and a lipophilic GEM prodrug, loaded with PFP. Under US, PFP undergoes liquid-to-gas transition, converting nanodroplets to microbubbles, increasing vascular permeability; the microbubbles then revert to nanoscale for interstitial penetration [5]. In acidic TME, DOPE-DMA hydrolyzes, causing neutral-to-positive charge inversion, enhancing transcytosis and tumor penetration, enabling efficient GEM release.

Lux et al. further showed that tumor acidosis can activate phospholipid-coated microbubbles (PC-MBs) for CEUS. PC-MBs exhibited approximately threefold increase in nonlinear harmonic signal when pH dropped from 7.4 to 5.5 due to acidity-induced changes in shell elasticity/interfacial tension [59]. The response was reversible and depended on phospholipid structure. This suggests that clinically used microbubbles (e.g., Lumason) may inherently serve as pH-responsive, tumor-selective US imaging agents without additional targeting modifications.

**Fig. 6:**
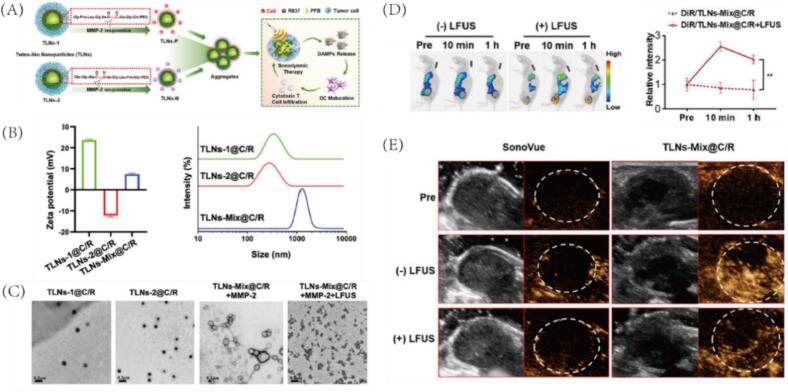
(A) Illustration of MMP-2-responsive TLNs co-transporting in circulation, aggregating at tumor sites for ultrasound imaging and sonodynamic therapy. (B) Zeta potential and size analysis of TLNs-1@C/R, TLNs-2@C/R, and TLNs-Mix@C/R after MMP-2 treatment (10 nM, 5 min, pH 6.5). (C) TEM images of TLNs with or without MMP-2 and LFUS exposure, showing morphological changes. (D) In vivo fluorescence imaging and quantification of tumor accumulation of DiR-labeled TLNs-Mix@C/R with or without LFUS at 8 h post-injection. (E) Representative B-mode and contrast-mode ultrasound images of tumors after intravenous injection of SonoVue or TLNs-Mix@C/R. Reproduced with permission from Ref.[9]© 2024 WILEY.

Matrix metalloproteinases (MMP-2/9) are highly expressed in tumor stroma and invasive margins, providing a biochemical trigger for enzyme-responsive nanoplatforms. To improve tumor uptake and retention, Shuai et al. developed an MMP-2-responsive “twin-like nanoparticle” (TLN) system composed of two nanoparticles (TLN-1@C/R and TLN-2@C/R) with complementary surface charges [9]. Each particle is constructed from MMP-2-cleavable block copolymers and co-loaded with Ce6, R837, and perfluorobutane (PFB) (Fig. 6A). Upon exposure to MMP-2 in the tumor microenvironment, the cleavable linkers are hydrolyzed, triggering a surface charge transformation that leads to in situ electrostatic self-assembly into large aggregates (∼1400 nm) (Fig. 6B). TEM confirmed the formation of these aggregates (Fig. 6C). In vivo, TLNs-Mix accumulated at tumor sites and, following LFUS irradiation at 8 h post-injection, showed a fivefold increase in DiR fluorescence, indicating enhanced tumor retention (Fig. 6D). Moreover, TLNs-Mix pretreated with MMP-2 exhibited significantly stronger ultrasound signals compared to untreated formulations. Unlike commercial SonoVue (∼2.5 μm), which failed to penetrate tumors and showed no US signal, TLNs-Mix produced robust US contrast, attributed to the formation of large, echogenic aggregates (Fig. 6E). This design ensures synchronized delivery of both nanoparticles and enhances tumor imaging and LFUS-mediated drug release through MMP-2-triggered in situ self-assembly and retention.

Tumors show high intracellular GSH (∼10 mM) versus low extracellular levels (∼2–10 μM), enabling redox-responsive targeted delivery. Sletten et al. constructed a disulfide-linked POx surfactant (P(MeOx)_30_-SS-P(NonOx)_10_) to stabilize PFOB nanoemulsions; surface −SS- bonds showed GSH-dependent cleavage, allowing reduction-triggered disassembly [47].

Exogenous triggers (e.g., laser) offer spatiotemporal control. Dong et al. developed NIR-absorbing NBF-based NPs co-loading DOX and PFCs (PDNBF). Upon 808-nm irradiation, photothermal heating induces PFC liquid-to-gas transition, enhancing ultrasound contrast and accelerating DOX release, achieving image-guided therapy [60].

Together, these stimulus-responsive strategies leverage the distinct biochemical features of the tumor microenvironment to achieve precise control over drug release, phase-transition behavior, and imaging contrast. By tailoring nanoplatforms to respond to specific endogenous or exogenous cues, these systems hold promise for improving the safety, specificity, and efficacy of ultrasound-mediated cancer theranostics. Although these functionalization strategies greatly enhance specificity, their increased structural complexity may raise immunogenicity risks and complicate GMP-compliant manufacturing, suggesting that future designs should prioritize minimal yet effective functional modules.

## 4 Ultrasound-guided cancer theranostic of PCCAs

Building on the above design principles, PCCAs have been extensively applied in ultrasound-guided cancer theranostics. Their tunable core–shell compositions and surface functionalities enable controllable acoustic activation, deep-tissue delivery, and microenvironment-responsive behavior in vivo. In this section, we first summarize ultrasound imaging modalities enabled by PCCAs and then discuss therapeutic strategies in which these phase-change platforms serve as central mediators of drug delivery, oxygenation, immune modulation, and other treatment paradigms.

### 4.1 Ultrasound imaging modalities enabled by PCCAs

PCCAs offer unique advantages for ultrasound imaging owing to their acoustic responsiveness, stability in circulation, and ability to undergo liquid-to-gas transformation under external stimuli. This section highlights various ultrasound imaging modalities enabled by PCCAs, emphasizing their roles in conventional contrast enhancement, vascular/morphological visualization, and microenvironment-responsive imaging.

#### 4.1.1 CEUS imaging

PCCAs significantly enhance CEUS imaging by enabling in situ microbubble formation that improves contrast sensitivity, vascular delineation, and tumor detectability. Although traditional microbubble-based contrast agents, such as SonoVue, have improved ultrasound imaging to a certain extent, their clinical utility remains limited due to poor stability, short circulation time, and inadequate tissue penetration. To address these limitations, a novel nanoscale liquid–gas phase-change red blood cell-mimicking contrast agent, termed Sonocyte, was developed [61]. Sonocyte is composed of uniform lipid-based nanodroplets with dodecafluoropentane (DFP) as the volatile core and is cloaked with multilayered red blood cell membranes (RBCm) (Fig. 6A). The formulation employs FDA-approved lipids (HSPC, cholesterol, and DSPE-mPEG_2K_), and the RBCm coating significantly enhances biocompatibility, prolongs systemic circulation, and promotes accumulation in target tissues, while reducing nonspecific cellular uptake. Notably, the low boiling point of DFP (29°C) enables efficient ultrasound-triggered phase transition at physiological temperature (37°C), ensuring robust acoustic responsiveness. Both in vitro and in vivo evaluations demonstrated that Sonocyte exhibits excellent stability, ultrasound sensitivity, and phase-change responsiveness. It enables sensitive detection of normal liver parenchyma and necrotic tissue and, importantly, accumulates effectively within solid tumors, achieving high sensitivity and specificity in tumor imaging, addresses the limitations of SonoVue in this regard. In summary, Sonocyte represents the first nanoscale RBC-functionalized phase-change contrast agent that meets multiple requirements for ultrasound imaging and holds great promise for clinical translation across a range of pathological conditions.

In another research, a phase-change nanoplatform was developed by encapsulating iron oxide nanoparticles (IONPs) within the PFH core of emulsion nanodroplets [62]. To enable stable dispersion of IONPs in the fluorous PFH phase, their surface ligands were modified with perfluorononanoic acid (PFNA), rendering them fluorophilic. The nanodroplets were stabilized using phospholipids and designed to undergo ADV upon ultrasound irradiation. Among them, PFH acts as a phase-change agent that transforms into microbubbles under ultrasound, enhancing imaging contrast; IONPs lower the vaporization threshold by facilitating energy absorption and converting acoustic energy into heat; the PFNA coating ensures compatibility with the fluorous core, enabling uniform nanoparticle distribution. This synergy enhances ultrasound responsiveness and imaging sensitivity. In preclinical models, the IONP-loaded PFH nanodroplets accumulated in tumor tissue and generated detectable microbubbles upon ultrasound stimulation, demonstrating tumor-selective activation. This platform offers a promising strategy for ultrasound-guided diagnosis and therapy, combining enhanced contrast generation with site-specific responsiveness.

**Fig. 7:**
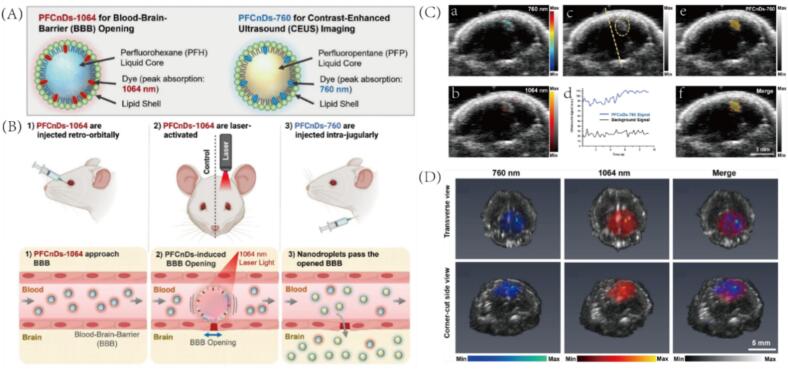
(A) Schematic of two PFCnDs with distinct optical absorbances (760 nm and 1064 nm), allowing multiplexed activation and imaging. Both have lipid shells for BBB opening and US/PA imaging. (B) Diagram of the intracerebral delivery method and underlying mechanism for delivering PFCnDs into the brain. (C(a, b)) PA/US coronal brain images at 760 nm and 1064 nm showing signal localization on the treated (right) side. US grayscale provides anatomical context. (C(c)) US B-mode image post 760  nm laser activation, revealing a bright echogenic area on the treated side. (C(d)) Graph of US signal intensity over time; laser stimulation increased signal from PFCnDs-760, but not from the control side. (C(e)) CEUS image using a detection algorithm highlights activated PFCnDs-760 regions (yellow) overlaid on the US map. (C(f)) Composite image showing co-localized PA, US, and CEUS signals on the treated brain region. (D) Volumetric US/PA images of an excised brain from a mouse treated with the intracerebral imaging and delivery approach. The mouse received intravenous injection of PFCnDs-760 and PFCnDs-1064 and was treated with PFCnD-induced BBB opening. Reproduced with permission from Ref.[63] © 2024 Springer Nature.

In a separate study, Stanislav Y. Emelianov et al. developed an innovative nanocontrast agent system for tracking drug delivery in the brain and enabling transcranial US/PA imaging [63]. They designed two types of perfluorocarbon nanodroplets (PFCnDs), both encapsulated with lipid membranes but incorporating different core materials (Fig. 7A). The first, PFCnD-1064, utilizes PFH as the core and is loaded with a material that strongly absorbs 1064  nm laser light, enabling localized and controllable opening of the BBB. Upon intracerebral injection, these droplets accumulate in the cerebral microvasculature near the BBB and, under laser stimulation, undergo cavitation to transiently open the barrier (Fig. 7B). The second formulation, PFCnD-760, contains PFP and a chromophore absorbing at 760  nm, designed for photoacoustic imaging. After BBB disruption, PFCnD-760 crosses into brain parenchyma (Fig. 7B). Due to the low boiling point of perfluoropentane, the nanodroplets exhibit a low vaporization threshold under physiological conditions. Upon activation, they efficiently convert into stable microbubbles, enabling prolonged ultrasound contrast enhancement. Transcranial US/PA imaging demonstrated that signals at both 760  nm and 1064  nm were localized to the treatment side of the brain, where a bright echo region was observed (Fig. 7C(a-c)). In contrast, minimal signal was detected on the control side. Notably, the ultrasound intensity of the PFCnDs-760 within the echo region on the treated side exhibited a gradual increase over time, indicating sustained accumulation or activation (Fig. 7C(d)). And the US and PA signals can achieve a good overlap (Fig. 7C(e-f)). These findings were further validated by in vitro experiments, which corroborated the in vivo imaging results (Fig. 7D). Collectively, these results highlight that through precise tuning of the two nanodroplet formulations, the researchers developed a multifunctional contrast agent platform capable of crossing the BBB, enabling deep brain delivery, and supporting real-time transcranial imaging.

Across these imaging studies, PCCAs consistently outperform conventional microbubbles in stability, tissue penetration, and activation controllability, yet their benefits vary strongly with core volatility and shell architecture, underscoring the need for standardized acoustic parameters when comparing performance.

#### 4.1.2 Tumor vasculature and morphological imaging

The acoustic responsiveness and stability of PCCAs enable high-resolution visualization of tumor vasculature, supporting assessment of angiogenesis and perfusion dynamics. Building upon this principle, Ellegala et al. investigated the feasibility of using CEUS with α_v_β_3_-integrin–targeted microbubbles to noninvasively image tumor angiogenesis [64]. They constructed PFC microbubbles conjugated with echistatin, a ligand that selectively binds α_v_β_3_ integrins highly expressed on angiogenic endothelium. In a rat glioma model, CEUS imaging at 14 and 28 days post-implantation revealed that these targeted microbubbles preferentially adhered to tumor neovessels, particularly at the periphery where integrin expression and angiogenic activity were most pronounced. By combining targeted imaging with perfusion measurements using non-targeted microbubbles, the study provided a comprehensive assessment of both molecular and hemodynamic aspects of tumor vasculature. Over time, an increase in microvascular blood volume was observed, despite a concurrent reduction in blood velocity, indicating the maturation and functional inefficiency of the tumor microcirculation. Notably, signal intensity from α_v_β_3_-targeted microbubbles correlated strongly with microvascular blood volume, underscoring the specificity of the targeting strategy. This work highlights the potential of ligand-directed PFC microbubbles in CEUS as a dual-purpose tool—both for detecting early angiogenic changes and for characterizing vascular function within tumors.

**Fig. 8:**
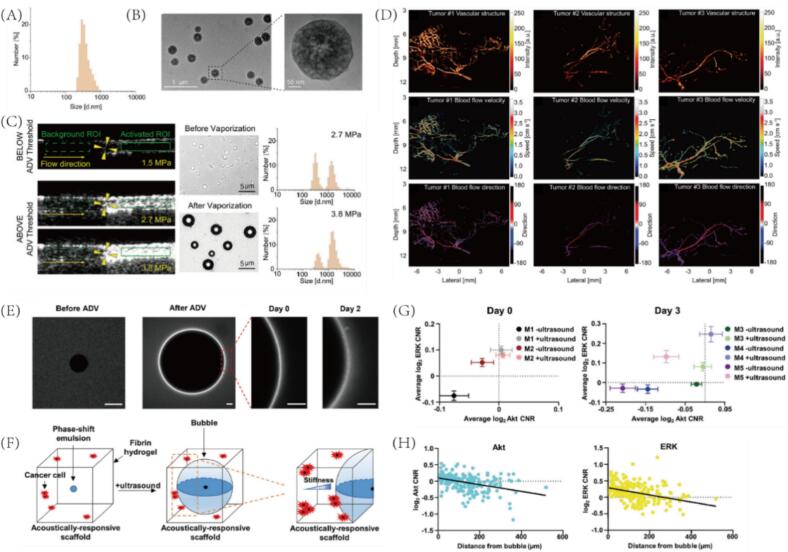
(A) DLS analysis shows PCND size distribution. (B) TEM images confirm PCND morphology. (C) B-mode ultrasound images of PCNDs (2 × 10^9^/mL) under different acoustic pressures; ADV-induced signal enhancement is marked. Microscopy and DLS data illustrate size changes before/after ultrasound at 2.7 and 3.8 MPa. (D) SR-ALUSA imaging visualizes tumor vasculature and blood flow. (E) Confocal images of acoustic-responsive scaffolds (ARSs) before and after ADV; fibrin matrix labeled with Alexa Fluor 647. (F) Schematic of ARS mechanism: ultrasound triggers ADV, forming bubbles that compact the surrounding fibrin matrix. (G) Quantification of Akt and ERK signaling in breast cancer cells in ARSs with or without ultrasound (n ≥ 60 cells/group). (H) Signaling activity of Akt and ERK correlates with proximity to ADV-generated bubbles (n = 215 cells/group). (A-D) Reproduced with permission from Ref.[65] © 2024 Springer Nature. (E-H) Reproduced with permission from Ref.[66] © 2022 WILEY.

Advancing further, An et al. developed a novel imaging technique termed Arterial Labeling Ultrasound Subtraction Angiography (ALUSA), employing PFB-based nanodroplets and stabilized by a lipid shell composed of DSPC and DSPE-PEG_2K_ [65]. Additionally, Pluronic F68 was incorporated to modulate surface tension. The monodisperse nanodroplets were synthesized with an average diameter of 381 nm (Fig. 8A), and transmission electron microscopy (TEM) revealed their well-defined core–shell spherical morphology (Fig. 8B). Upon ultrasound exposure exceeding 2.38 MPa, the nanodroplets underwent a liquid-to-gas phase transition, generating microbubbles for enhanced imaging contrast (Fig. 8C). By directing focused ultrasound to a specific vessel, ALUSA selectively activates nanodroplets at the labeling site and dynamically tracks downstream perfusion using plane-wave imaging. In preclinical models, including rabbit kidney and murine breast tumors, ALUSA achieved super-resolution visualization (up to 36  μm), enabling detailed mapping of microvascular networks and quantification of flow velocity and direction (Fig. 8D). Compared to conventional CEUS, which enhances all perfused vessels indiscriminately, ALUSA provides high spatial specificity by isolating signal enhancement to the selected arterial supply. Moreover, this method is noninvasive, repeatable, and free from ionizing radiation or nephrotoxic agents, offering a safer and more targeted alternative for assessing tumor perfusion. These findings underscore the versatility and translational potential of PFC-based nanodroplets in high-resolution functional ultrasound imaging and mark an important advance toward precision vascular diagnostics in oncology.

Cancer cells continuously sense and respond to mechanical cues from the extracellular matrix (ECM), and interactions with the ECM can remodel intracellular signaling cascades, influencing processes such as proliferation, migration, and survival. Gary D. Luker et al. employed a recently developed composite hydrogel system termed the ARSs, composed of a fibrin matrix and PFC emulsions [66]. Results indicated that ADV under focused ultrasound stimulation causes the fibrin matrix around the bubbles to coagulate, thereby locally enhancing the fluorescence signal within the matrix (Fig. 8E). Changes in the fibrin matrix further affect the signal transmission of triple-negative breast cancer (TNBC) cells encapsulated in ARSs (Fig. 8F). Among them, they found that the activity of two key oncogenic kinases, Akt (protein kinase B) and ERK (extracellular signal-regulated kinase), under both basal and growth factor-stimulated conditions was negatively correlated with the distance to ADV-induced bubbles (Fig. 8G-H). These observations were validated in both in vitro cultures and in murine models. In summary, the PFC-mediated phase transition and ultrasound-guided spatial control provide a precise and dynamic approach to interrogate how local ECM densification regulates cancer cell signaling. The ARSs platform offers a powerful tool to dissect the spatiotemporal biomechanical influences of the tumor microenvironment, with promising implications for cancer biology research and the development of therapeutic strategies.

### 4.2 Therapeutic strategies facilitated by PCCAs

#### 4.2.1 Ultrasound-mediated monotherapy

**Fig. 9:**
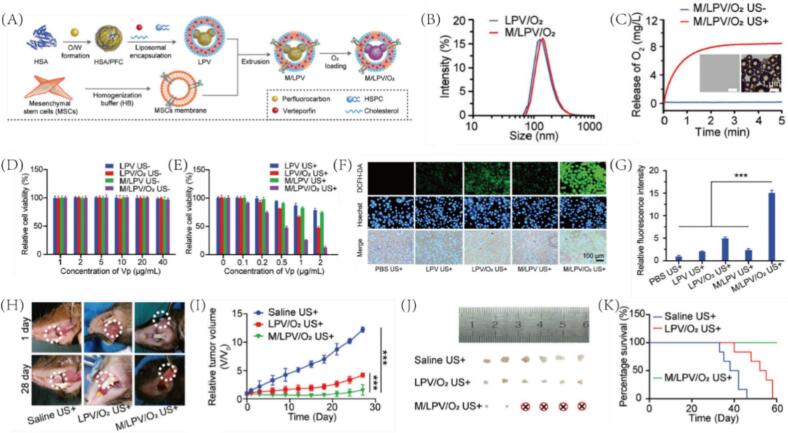
(A) Schematic of M/LPV/O_2_ nanoparticle preparation. (B) Size distribution of LPV/O_2_ and M/LPV/O_2_ formulations. (C) O_2_ release and ultrasound-induced microbubble formation (±US, 0.35 W/cm^2^, 1 MHz, 3 min). (D) Cell viability of Cal-27 cells without US treatment. (E) Cell viability of Cal-27 cells under US treatment. (F) Intracellular ROS fluorescence in Cal-27 cells after different treatments with US. (G) Quantification of intracellular ROS levels. (H) In vivo fluorescence imaging of tumors post-treatment (saline, LPV/O_2_, M/LPV/O_2_ + US). (I) Tumor growth curves under different treatments (n = 6). (J) Images of tumors after 28 days of treatment. (K) Survival analysis of mice with orthotopic oral tumors under different treatments + US. Reproduced with permission from Ref.[68] © 2020 WILEY.

PCCAs enhance ultrasound monotherapies such as sonodynamic therapy (SDT) by improving sensitizer activation, ROS generation, and microbubble-assisted mechanical disruption [67]. To overcome the limitations of conventional treatments for oral squamous cell carcinoma (OSCC), such as invasive injury, impaired oral function, and unsatisfactory efficacy, Sun et al. developed a biomimetic sonodynamic nanoplatform (M/LPV/O_2_), constructed from oxygen-loaded polydopamine nanoparticles, the sonosensitizer verteporfin (Vp), and liposomes, with surface modification by mesenchymal stem cell (MSC) membranes to enhance tumor targeting[68] (Fig. 9A). The nanoparticles (∼150 nm) rapidly release oxygen upon 1 MHz ultrasound exposure, generating abundant microbubbles within 3 min (Fig. 9B-C). In Cal-27 cells, M/LPV/O_2_ showed strong ultrasound-triggered cytotoxicity and good biosafety without stimulation (Fig. 9D-E). Under hypoxia, it significantly elevated ROS levels compared to LPV and M/LPV, indicating effective oxygen delivery for enhanced SDT (Fig. 9F-G). Fluorescence analysis showed its cellular uptake was ∼3-fold higher than LPV/O_2_, highlighting the role of MSC membranes (Fig. 9F). In an orthotopic OSCC mouse model, M/LPV/O_2_ achieved robust tumor inhibition, prolonged survival, and preserved oral and facial integrity (Fig. 9H-K). This biomimetic nanoplatform shows great promise for clinical sonodynamic cancer therapy.

*Chen* et al. constructed cationic porphyrin-loaded NPs using fluorocarbon/PEG block copolymers (RAFT) [69]. The fluorocarbon core provided efficient oxygen-carrying capacity to relieve tumor hypoxia, while the cationic shell improved uptake. Porphyrin functioned as the ultrasound-activated ROS generator. This PFC-based nanosystem markedly increased intratumoral O_2_ and ROS during SDT, inducing strong apoptosis in Renca tumors, and also offered useful imaging contrast. Such oxygen-enhanced PFC carriers can further help overcome hypoxia-associated resistance in SDT and other therapies.

#### 4.2.2 Combination with chemotherapy

Monotherapy faces translational limits, motivating PCCA-based combination strategies. In TNBC, chemo or gas therapy alone is insufficient. A natural pollen-derived, US-responsive carrier was developed to co-deliver DOX and PFCs [70]. The hollow pollen structure loads oxygen-rich PFCs, while the porous shell adsorbs DOX. Under ultrasound, PFCs release oxygen and DOX acts as both a chemotherapeutic and a sonosensitizer, jointly boosting intratumoral oxygenation and ROS generation. In vivo, this PO/D-PGs system markedly suppressed TNBC growth, underscoring the potential of PFC-based pollen microcarriers for enhanced chemo–sonodynamic therapy.

**Fig. 10:**
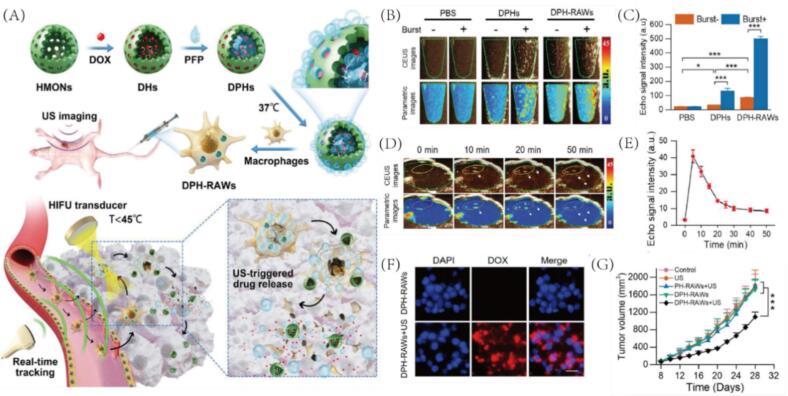
(A) Schematic illustration of the macrophage-mediated drug delivery system. (B) In vitro contrast-enhanced ultrasound (CEUS) and parametric images of agar phantoms containing PBS, DPHs, or DPH-RAWs, captured before and after single-pulse sonication (24 MHz, 100 % power, 1 s). Bright spots indicate microbubble formation (n = 6) and (C) Quantitative echo intensity analysis. (D) In vivo CEUS and parametric images of 4 T1 tumors before and after DPH-RAWs injection and (E) time-course echo intensity quantification. (F) Fluorescence imaging and cell viability of 4 T1 cells after 24 h incubation with media from irradiated or non-irradiated DPH-RAWs. (G) Tumor growth curves of 4 T1-bearing mice under different treatments (n = 5). Reproduced with permission from Ref.[11] © 2020 WILEY.

Expanding on the theme of cellular carriers, Yan et al. developed an ultrasound-activated “cell bomb” system for targeted drug delivery [11]. DOX and phase-changeable perfluoropropane (PFP) were co-loaded into hollow mesoporous organosilica nanoparticles (HMONs), forming DOX/PFP-loaded nanoparticles (DPH) (|Fig. 10A). These were subsequently internalized by RAW 264.7 macrophages to construct the cell-based delivery platform (DPH-RAWs). Upon exposure to high-intensity ultrasound, both DPD and DPD-RAWs rapidly generated microbubbles, as confirmed by ultrasound imaging (Fig. 10B-C). In vivo, intratumoral injection of DPD-RAWs into 4 T1 tumor-bearing mice led to a strong contrast signal within tumor vasculature, which intensified over 20 min, indicating effective PFP vaporization (Fig. 10D-E). Furthermore, ultrasound triggered the intracellular release of DOX, resulting in significant cytotoxicity against 4 T1 cells and marked tumor growth inhibition in vivo (Fig. 10F-G). This study presents a promising strategy for macrophage-mediated, ultrasound-responsive drug delivery with real-time imaging capability and therapeutic precision.

Tumor hypoxia poses a significant barrier to the efficacy of oxygen-dependent therapies such as chemotherapy and sonodynamic therapy. Consequently, Zhao et al. further addressed the hypoxia barrier in pancreatic cancer by designing a novel microfluidic microcapsule delivery system [71]. This system featuring a perfluorocarbon core for oxygen storage and release, and a hydrogel shell co-loaded with the antimetabolite gemcitabine and the sonosensitizer indocyanine green, forming a composite platform named PO/GI-MC. The results demonstrated that PO/GI-MC effectively alleviated tumor hypoxia, increased ROS generation during SDT, and enabled Gem release under low-intensity ultrasound, thereby inducing synergistic cytotoxic effects. Notably, PO/GI-MC achieved significantly enhanced antitumor efficacy in patient-derived pancreatic cancer organoid models. These findings suggest that this oxygen-loaded microcapsule platform holds great potential as an effective strategy to overcome tumor hypoxia and augment the therapeutic performance of chemo-sonodynamic therapy.

#### 4.2.3 Synergistic photothermal and photodynamic therapy

Beyond chemotherapy, PCCAs have also been engineered for synergistic PTT/PDT to overcome tumor hypoxia. Li et al. reported a NIR-II responsive PCCA (PSPP-Au980-D) with a PFC core for O_2_ loading, a Si-phthalocyanine PDT component, and a PNIPAM shell bearing 980-nm AuNRs and DOX [14]. Under 980-nm irradiation, AuNR-mediated heating triggers O_2_ and DOX release, while 680-nm light activates PDT. Operating in the NIR-II window enables deep-tissue photothermal conversion and PA imaging. The combined PDT, PTT, and chemotherapy constitute a programmed cascade therapy strategy that significantly suppresses orthotopic pancreatic tumor growth.

**Fig. 11:**
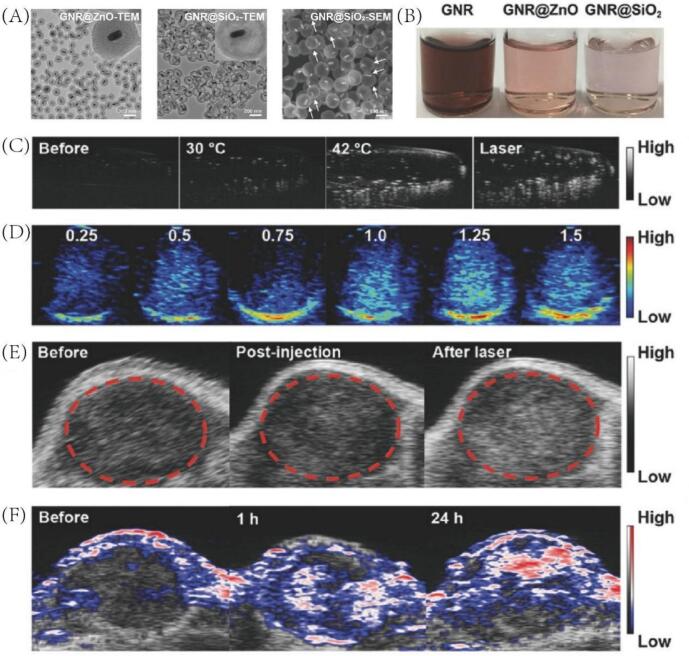
(A) TEM or SEM images of GNR@ZnO and GNR@SiO_2_. (B) Photograph of GNR, GNR@ZnO, and GNR@SiO_2_ aqueous solutions. (C) In vitro US images of GNR@SiO_2_-PFP solutions (10 ppm of Au) before heating, after heating (30 and 42C), and after laser irradiation (1 W cm^−2^, 5 min). (D) In vitro PA images of GNR@SiO_2_-PFP solutions at various concentrations with different OD values (0.25–1.5). (E) In vivo US images of tumor tissues before injection, 24 h postinjection of GNR@SiO_2_-PFP, and after laser irradiation (1 W cm^−2^, 5 min). (F) In vivo PA images of tumor tissues in mice at different time before and after injection of GNR@SiO_2_-PFP. Reproduced with permission from Ref.[72] © 2017 WILEY.

In a parallel approach, Huang et al. developed a nanoplatform with a gold nanorod (GNR) core and a thin mesoporous silica shell, encapsulating PFP, referred to as GNR@SiO_2_-PFP [72] (Fig. 11A-B). Upon NIR laser irradiation, the photothermal effect generated by the GNR inducing the liquid-to-gas phase transition of encapsulated PFP and thereby generating microbubbles that significantly enhance the US imaging signal (Fig. 11C-D). Furthermore, the GNRs feature tunable localized surface plasmon resonance around 800 nm, endowing the nanoplatform with strong PA imaging capabilities (Fig. 11E-F) and potent PTT effects. Importantly, the thin-shell mesoporous silica architecture provides a larger internal cavity, enhanced structural flexibility, and superior biodegradability compared to conventional dense silica nanoparticles, thus greatly improving PFP loading capacity and biosafety. Taken together, the GNR@SiO_2_-PFP nanoplatform enables enhanced US/PA dual-modality imaging-guided photothermal therapy for melanoma, demonstrating significant potential for integrated cancer theranostics.

#### 4.2.4 Integration with immunotherapy

**Fig. 12:**
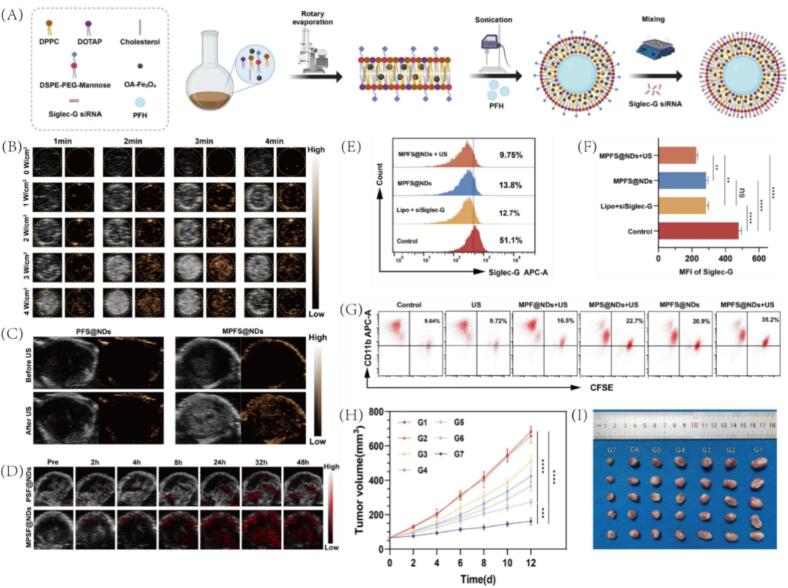
(A) Appearance of MPFS@NDs. (B) In vitro US imaging performance under different irradiation time and power. (C) In vivo US imaging performance after PFS@NDs and MPFS@NDs treatment. (D) Photoacoustic images of the tumor site in 4 T1 tumor-bearing mice at different time points after injection of MPFS@NDs or PFS@NDs. (E) FCM analyses of Siglec-G expression in different groups of macrophages. (F) Significant difference analysis of Siglec-G expression in different groups of macrophages. (G) Phagocytosis of 4 T1 tumor cells by macrophages treated with MPFS@NDs combined with US. (H) Tumor growth curves of different groups, n = 5, mean ± SD. (I) Digital photos of harvested tumors; n = 5. Results are presented as mean ± SD. Reproduced with permission from Ref.[13] © 2024 Springer Nature.

Given the central role of the tumor immune microenvironment in disease progression and therapy resistance [73], integrating PCCAs with immunotherapy has opened new avenues for reshaping immune responses and enhancing treatment efficacy. TNBC is characterized by an immunosuppressive tumor microenvironment, where the phenotype and function of tumor-associated macrophages (TAMs) play a pivotal role in disease progression. To overcome this barrier, Nie et al. developed a theranostic, ultrasound-responsive nanoplatform, termed MPFS@NDs [13]. This system comprises lipid nanodroplets encapsulating PFH as a phase-change core, embedded with Fe_3_O_4_ nanoparticles, and surface-modified for selective targeting of M2-type TAMs (Fig. 12A). Additionally, the nanodroplets deliver Siglec-G siRNA to disrupt the CD24/Siglec-G “don’t eat me” signaling pathway (Fig. 12A). Upon ultrasound activation, MPFS@NDs undergoes ADV, transitioning into microbubbles and enhancing US signal intensity in a time- and power-dependent manner (Fig. 12B). In 4 T1 tumor models, US-triggered MPFS@ND45 showed significantly enhanced US and PA imaging signals, confirming effective tumor accumulation and phase transition (Fig. 12C-D). Functionally, MPFS@NDs + US treatment resulted in the greatest reduction of Siglec-G^+^ macrophages, demonstrating efficient siRNA delivery and gene silencing (Fig. 12E-F). This was accompanied by enhanced phagocytosis of tumor cells by M2 macrophages (Fig. 12G). Under such an immunomodulatory strategy, effective tumor suppression was achieved (Fig. 12H-I). Moreover, the treatment promoted CD8^+^ T cell activation and infiltration, leading to substantial inhibition of primary tumor growth and lung metastasis. Overall, this multifunctional, non-viral delivery platform offers potent therapeutic efficacy and multimodal imaging capability, showing strong promise for clinical translation in cancer therapy.

A PFC-based platform (UCNP@mSiO_2_-PFC/Ce6@RAW-Man/PTX) integrates 808-nm-responsive upconversion activation of Ce6 with PFC-mediated oxygen delivery and mannose-modified macrophage-membrane targeting [74]. The Er/Lu core–shell UCNP converts NIR to 660 nm to trigger PDT, while PFC alleviates hypoxia and enhances photoacoustic O_2_ readout. The macrophage-membrane coating improves TAM targeting and supports M2 to M1 repolarization, with low-dose PTX further boosting immunomodulation. Both in vitro and in vivo studies show enhanced cytotoxicity and tumor inhibition, illustrating the synergistic PDT-oxygenation-immune effects of this PFC-enabled system.

#### 4.2.5 Other multimodal therapeutic approaches

Starvation therapy, which deprives tumor cells of essential nutrients such as glucose, has emerged as a promising approach for metabolic disruption in cancer treatment. However, its efficacy is often limited by tumor hypoxia and the low catalytic efficiency of endogenous enzymes. To overcome these barriers, recent studies have explored multimodal platforms that integrate starvation therapy with other therapeutic modalities and imaging techniques[75], [76]. For instance, Zhou et al. developed an ultrasound-responsive, visualizable liposomal nanoplatform—tLyP-1H(Gd)-GOD@PFP (THGP)—for imaging-guided synergistic starvation and sonodynamic therapy [12]. The platform incorporates GOD, H(Gd), and PFP, and is surface-functionalized with the tLyP-1 peptide to enhance tumor targeting and deep tissue penetration. GOD consumes glucose and generates H_2_O_2_, inducing energy deprivation while cooperating with ultrasound-activated H(Gd)-derived ^1^O_2_ to amplify oxidative stress. Simultaneously, PFP undergoes phase transition under low-intensity focused ultrasound (LIFU), releasing oxygen to alleviate hypoxia and accelerate catalytic reactions. MRI and PAI enable real-time tracking of nanoparticle accumulation, while USI monitors cavitation and oxygen release, allowing precise control of treatment timing. The platform significantly enhances ROS production (3.3-fold), increases the catalytic rate (1.5-fold), and improves oxygen delivery (2.3-fold), resulting in marked tumor suppression in vivo with good biosafety. This study highlights the potential of combining nanomedicine and ultrasound imaging for precision cancer therapy.

A mesoporous polydopamine-based system (MGPO NPs) integrates PFP and GOx to enable US/PAI-guided synergistic PTT and enhanced starvation therapy. NIR-triggered PFP liquid–gas transition improves US contrast, while MPDA provides PAI for tumor localization [77]. PTT induces ablation and promotes PFP-derived O_2_ release, relieving hypoxia and augmenting GOx-mediated glucose depletion into H_2_O_2_ /acid. In CT-26 models, MGPO achieved complete tumor inhibition with minimal toxicity, with real-time US/PAI tracking confirming precise delivery and oxygenation changes, representing a typical PFC-assisted multimodal theranostic strategy. Moreover, advances in NIR-II absorbing organic nanoagents provide robust PA and photothermal modules that can be integrated with PFC-based oxygen reservoirs to realize deep-tissue multimodal theranostics [78].

Beyond classical apoptosis, emerging programmed cell death (PCD) pathways such as ferroptosis and cuproptosis have drawn increasing attention for their potential to overcome tumor resistance. Ferroptosis involves lipid peroxidation driven by iron metabolism, while cuproptosis is triggered by copper-induced mitochondrial stress and Fe–S cluster protein destabilization. However, their efficacy is often constrained by tumor hypoxia and limited ROS generation. Recent studies couple PCD pathways with SDT using oxygen-releasing PFC platforms to enable imaging-guided synergistic therapy [79]. Ning et al. designed a biomimetic PFC-core nanosystem (SAFE) integrating SDT with ferroptosis induction [80]. SAFE uses oxygen-carrying PFC loaded with DGLA and VP, camouflaged with RBC membrane modified by CD47/iRGD for prolonged circulation and tumor targeting. After tumor accumulation, ultrasound triggers payload release: PFC boosts local O_2_ to enhance VP-mediated ROS under SDT, while ROS simultaneously drives DGLA lipid peroxidation, promoting ferroptosis. This strategy demonstrates PFC-based PCCAs can provide ultrasound imaging and dual SDT–ferroptosis therapy with synergistic antitumor efficacy.

Building upon their ferroptosis-based strategy, they further explored copper-induced cytotoxicity by developing a biomimetic nanorobot, SonoCu, thereby expanding their platform to target cuproptosis via mitochondrial dysfunction. In this research, they developed a novel cell-membrane-biomimetic nanorobot, termed SonoCu, which integrates SDT with copper-induced cytotoxicity to achieve multimodal therapeutic synergy [81]. The formulation of SonoCu involves the construction of a copper-doped ZIF-8 framework encapsulating PFC and the sonosensitizer Ce6, and camouflaged with macrophage membranes, SonoCu achieves tumor-targeted delivery and long circulation. The ZIF-8 structure serves as the pH-responsive component, dissociating in the acidic tumor microenvironment to trigger the release of Cu^2+^ and Ce6, a process further enhanced by ultrasound stimulation. This dual pH/ultrasound responsiveness enables spatiotemporally controlled drug release, improving both therapeutic precision and potential for dynamic metabolic imaging. PFC, upon ultrasound activation, releases oxygen to alleviate tumor hypoxia, thereby enhancing ROS generation and imaging contrast. Moreover, copper toxicity disrupts mitochondrial metabolism by downregulating Fe-S cluster proteins such as FDX1 and LIAS, providing possible targets for metabolic imaging. The macrophage membrane coating facilitates tumor accumulation and immune evasion, offering a high signal-to-noise foundation for responsive imaging. Collectively, SonoCu exemplifies a sophisticated multimodal platform with synergistic therapeutic and imaging capabilities, laying a foundation for real-time, microenvironment-adaptive cancer theranostics.

Taken together, oxygen-supplemented SDT platforms show the most consistent therapeutic enhancement across tumor models, whereas combination regimens incorporating chemotherapy or phototherapy offer higher potency but also greater formulation complexity.

## 5 Conclusion

Over the past decade, liquid–gas phase-change nanoplatforms have emerged as a transformative class of ultrasound contrast agents, enabling precise tumor imaging and multifunctional therapeutic interventions. By leveraging the unique acoustic-responsive phase transition of PFC-based nanostructures, PCCAs offer enhanced stability, deeper tissue penetration, and controllable activation, addressing the inherent limitations of conventional microbubble-based UCAs. Advances in core material selection, encapsulation strategies, and surface functionalization have significantly expanded the versatility of PCCAs for ultrasound-guided cancer theranostics. In particular, the integration of targeting ligands and stimulus-responsive elements has enabled tumor-selective accumulation, real-time monitoring, and controlled release of therapeutic payloads, facilitating a more precise and personalized approach to cancer management.

## 6 Challenges and future perspectives

Despite substantial progress, the translation of PCCAs into clinical use is still constrained by challenges related to scalability, GMP-compliant manufacturing, immunogenicity, and regulatory approval. Current fabrication techniques, including microfluidic emulsification and solvent evaporation, offer excellent control over droplet size and composition, while simultaneously encountering difficulties in maintaining batch-to-batch reproducibility and compliance with GMP standards during scale-up. In parallel, the immunogenic potential of shell materials and surface coatings remains insufficiently characterized, necessitating systematic evaluation of complement activation, long-term biodistribution, and clearance pathways. From a translational standpoint, the absence of defined regulatory guidelines for nanoscale phase-change agents poses further uncertainty, highlighting the need for standardized protocols aligned with existing ultrasound contrast agents. Furthermore, integration of PCCAs into clinical ultrasound systems demands harmonized acoustic parameters and validated safety thresholds. Addressing these interconnected issues through collaborative efforts in materials design, manufacturing science, and regulatory policy will be essential to pave the way for clinical realization of ultrasound-mediated nanotheranostics.

Looking forward, the future development of PCCAs will likely benefit from interdisciplinary convergence across materials science, acoustic physics, molecular biology, and clinical medicine. Smart design strategies that enable multi-level responsiveness, logic-gated release, and multimodal imaging hold promise for advancing the next generation of ultrasound-guided nanotheranostics. Moreover, the integration of artificial intelligence and machine learning into ultrasound data analysis may further enhance diagnostic precision and therapy monitoring. With growing synergy across disciplines and a clear path toward clinical translation, PCCAs are set to reshape the landscape of noninvasive cancer management through intelligent ultrasound-guided theranostics.

## CRediT authorship contribution statement

**Jin Lei:** Writing – original draft, Investigation, Formal analysis, Data curation. **Jing Lin:** Writing – review & editing, Supervision, Funding acquisition, Conceptualization. **Peng Huang:** Writing – review & editing, Supervision, Funding acquisition, Conceptualization.

## Declaration of competing interest

The authors declare that they have no known competing financial interests or personal relationships that could have appeared to influence the work reported in this paper.
